# Exploring the Confluence of Animal Medicine and its Implications for Human Health: A Systematic Literature Review

**DOI:** 10.2174/011570159X333443240822115028

**Published:** 2024-09-16

**Authors:** Josie Dunn, Fabrizio Schifano, Ed Dudley, Amira Guirguis

**Affiliations:** 1 Medical School, The Grove, Swansea University, Swansea, SA2 8PP, UK;; 2 Psychopharmacology, Drug Misuse and Novel Psychoactive Substances Research Unit, School of Life and Medical Sciences, University of Hertfordshire, Hatfield, AL10 9AB, UK

**Keywords:** Veterinary medicines, animal medicines, substance use, diversion of medicines, drug misuse, self-medication

## Abstract

**Introduction:**

The abuse of veterinary drugs has emerged as a concerning trend, with global fatalities on the rise. Our understanding of this phenomenon remains limited. This study aims to identify the veterinary drugs being misused, the reasons behind their misuse, and how they are obtained.

**Methods:**

Utilising PubMed, Scopus, and Web of Science databases, along with related grey literature, we applied the Preferred Reporting Items for Systematic Reviews and Meta-analyses (PRISMA) framework for data collection. Screening and cross-referencing yielded 66 relevant articles, encompassing case reports, surveys, reports, and systemic literature reviews. The analysis identified 28 distinct veterinary drugs being misused in humans, primarily falling into categories, *e.g*., α-2- and β-2-adrenergic receptor agonists, GABAergic receptor modulators, opioid receptor agonists, non-steroidal anti-inflammatory drugs (NSAIDs), and N-methyl-D-aspartate (NMDA) receptor antagonists. These drugs were used for various purposes, including recreational use, weight loss, bodybuilding, pain relief, and self-medication for stress-related symptoms.

**Results:**

Routes of administration predominantly included parenteral, oral, and inhalation methods. Veterinary workers/assistants and individuals connected to animals were identified as contributors to the misuse of these medications. Motivations for their utilisation ranged from affordability and accessibility to the ease of obtaining multiple prescriptions from various veterinary sources, often in conjunction with other illicit substances. Dependence and addiction were common outcomes of the misuse of veterinary medicines by humans.

**Conclusion:**

Overall, this systematic review underscores the increasing popularity of veterinary prescription drug misuse despite being under-reported with limited available data. Healthcare professionals are urged to remain vigilant to potential overdose events involving these medications.

## 1 INTRODUCTION

As the global crisis of prescription drug misuse continues to escalate, individuals grappling with substance use are relentlessly seeking new avenues to satisfy their cravings. According to the National Survey on Drug Use and Health (NSDUH), diversion of prescription medicines is defined as 'use without a prescription or in ways not intended by the prescriber' [1]. This issue has been starkly characterised as a “public health disaster, killing hundreds of people and ruining the lives of millions” by Harry Shapiro, Head of the addiction charity DrugWise, during a 2016 meeting of the All-Party Parliamentary Group for Prescribed Drug Dependence [2].

In 2022-23, the expenditure on prescription items dispensed in England reached £10.4 billion, reflecting a 3% increase from the previous year [3]. Among the array of prescription drugs subject to abuse are opioids, benzodiazepines, stimulants, antidepressants, and steroids. The mounting misuse of these medications has prompted medical professionals and policymakers to label it a global health crisis, with misuse reaching alarming proportions [4]. In the United Kingdom (UK), deaths associated with codeine and tramadol surged by over fivefold among males and nearly eightfold among females between 1998 and 2021. Moreover, opioids were implicated in approximately 50% of drug poisoning cases reported in the UK in 2021, accounting for 45.7% of cases, equating to 2219 deaths [5].

In an increasingly interconnected world, the diversion of veterinary and human medicine is gaining prominence as a pivotal focal point. Veterinarians, who annually treat numerous animals and have the authority to prescribe controlled substances, are often overlooked as potential contributors to prescription drug misuse [6]. A survey conducted in 2023 explored the perspectives of UK veterinarians regarding the potential misuse of veterinary prescription medications (VPMs). The findings revealed that 88% of participants recognised the risk of abuse associated with certain VPMs. Furthermore, 30% of respondents reported suspicions of pet owners misusing VPMs, while 20% expressed concerns about misuse among veterinary staff [7]. The growing inclination towards acquiring medications through healthcare providers, such as veterinarians, is a familiar trend owing to the perception of these drugs being safer than those obtained through illicit channels, as well as being more cost-effective [8]. Additionally, the purchase of veterinary medicines online in the UK is reportedly on the rise [9]. The practice of “vet shopping” involves soliciting veterinarians for prescription medications intended for animals without the intention of administering them to the animals in question [10]. This behaviour significantly contributes to the escalating global issue of substance misuse as individuals gain access to additional drug supplies through veterinarians. A study conducted in 2022 revealed a threefold increase between 2014 and 2019 in the number of clients obtaining prescriptions for any class of controlled substances from four or more veterinarians [11]. The surge in acquiring medications through veterinarians prompted the United States Food and Drug Administration (US FDA) to express concerns in 2018, highlighting the significant risk posed by the prescription of opioids by veterinarians. Like opioid medications intended for human use, these drugs hold the potential for addiction, abuse, and overdose when diverted for personal use [12]. News articles have reported novel methods employed by individuals to access these controlled substances, such as harming their pets to obtain analgesics [13] and training their dogs to simulate symptoms to receive hydrocodone cough syrup [14].

The issue extends beyond the misuse of prescription drugs approved for human use; there has been a concerning increase in the misuse of medications exclusively approved for animal use. This trend is alarming as drugs approved solely for animal use have not undergone testing on humans, potentially resulting in a range of adverse effects due to anatomical, physiological, and pharmacokinetic differences. Unlike in human development, pre-clinical trials for animal medicine are not necessarily utilised, meaning human safety is not a focus [15]. The administration of larger doses in animals, owing to variations in hepatic metabolism [16], increases the risk of toxic effects when these medications are misused in humans. For example, veterinary ketamine formulations can be ten times stronger than human formulations [17]. Recreational ketamine use and associated fatalities are on the rise [18], with the prevalence of ketamine use in the last year increasing by 3.8% [19]. Conversely, carfentanil, approved only for animal use due to its potency being 100 times higher than fentanyl [20], was the second most frequently reported synthetic opioid in the United States between 2016 and 2017 [21], prompting the World Health Organisation to declare it a serious threat to public health.

Given that prescription drug misuse in veterinary settings remains an underestimated and under-researched area [6], this study aims to enhance understanding regarding the types of veterinary medications that are misused, the intentions behind their misuse, and the methods of acquisition.

## 2 METHODOLOGY

A systematic review involves the meticulous analysis of well-defined research questions employing a systematic and explicit methodology to identify, select, and critically evaluate pertinent research, as well as to analyse the data derived from the studies incorporated [22]. To ensure objectivity and rigour in study selection, a systematic and structured approach was adopted. Preferred Reporting Items for Systematic Reviews and Meta-Analysis (PRISMA) guidelines [23] were adhered to, providing consistency and transparency in the collection of suitable studies, facilitating a clear and structured approach to data collection. In November 2023, a systematic search was conducted using PubMed (NCBI), Web of Science (Clarivate), and Scopus (Elsevier) databases. The aim was to identify the most appropriate scientific databases for this study. PubMed was praised for its convenience, speed, and user-friendliness, which is particularly significant for clinicians and researchers [24]. It also affirmed that Scopus covers a broader range of journals compared to PubMed and Web of Science. Additionally, it highlighted Google Scholar's utility in retrieving less mainstream information, albeit with the drawback of infrequent updates.

Boolean operators (AND/OR) were utilised to combine two groups of words into the final string utilised in all three databases. An iterative process of optimisation and refinement was utilised to ensure the retrieval of pertinent and comprehensive articles. Initially, various combinations of the search thread were explored to determine their effectiveness in capturing relevant literature. Further adjustments were made to the search strategy until a final search thread was determined. The string ((“veterinary drug” OR “veterinary medication” OR “veterinary prescription drug”) AND (“misuse” OR “abuse”)) was entered into the three scientific databases. We established clear inclusion and exclusion criteria to ensure a selection of papers relevant to our research questions and the aims of the study. Specifically, we included articles that addressed the misuse or diversion of veterinary medicine regarding human consumption. This encompassed literature reviews, case studies, and reports focusing on the unauthorised use, misuse, or non-medical consumption of veterinary drugs. Conversely, we excluded papers that did not explicitly reference the misuse or diversion of veterinary pharmaceuticals in humans. The risk of biased was assessed using the risk of bias in systematic reviews (ROBIS) tool [25], where each study was individually evaluated by JD and peer-reviewed by AG. A thematic approach was employed to analyse the existing literature. This type of analysis aided in the identification of specific themes present within the literature. Following a systematic review of all articles, the data was organised based on categories, including drug class, classification as human or animal drugs, and controlled substance status. The search was not restricted by time or geographical limitations, and all languages were included in the search results. Identification of grey literature was conducted between November and December 2023, involving examination of government reports and manual scrutiny of supplementary articles through Google Scholar. Microsoft Excel (Version 16.79.1 (23111614)) served as a tool to eliminate duplicate articles. A supplementary cross-reference search was conducted on the remaining studies to mitigate the risk of overlooking relevant articles in the systematic search.

## 3 RESULTS

Initially, a total of 338 records were identified, encompassing both database searches and various sources of grey literature. Following the completion of the screening process, 66 articles were found to be relevant to the current study and met all the points of the inclusion criteria. A total of 272 articles were excluded as they did not meet the inclusion criteria. Within this body of literature, 28 distinct veterinary drugs were identified as being misused by humans or posing a risk to human health. Fig. (**1**) provides a summary of the process through which records were identified, screened, and assessed for eligibility. Subsequently, each remaining article underwent further analysis, and the main findings from the selected articles and reports were summarised in Table (**S1**). This Table **S1** provides insight into the off-label use/ indication for each of the diverted veterinary medicines being identified in this literature review, the dose consumed, the routes of administration, and where each medicine was obtained.

One of the primary objectives of the systematic literature review was to identify the types of veterinary medications susceptible to misuse by humans or currently being misused. The primary classes of drugs identified included α-2- and β-2-adrenergic receptor agonists, NMDA antagonists, opioid receptor agonists, GABAergic receptor modulators, and non-steroidal anti-inflammatory drugs (NSAIDs). Table **1** provides a summary of the veterinary drugs obtained from the literature, along with the primary reasons for their misuse in humans.

Among the drugs identified, those approved exclusively for animal use constituted 53.6% of the total drugs retrieved from the literature (15 out of 28). The remaining 13 drugs were approved for both human and animal use, although some were administered at higher doses that were licensed for animal use only. Table **2** outlines the approved/licensed usage of each veterinary drug identified and its legal classification in both the UK and the US.

Among all medicines identified as misused by humans, 68% (19 out of 28) are not classified as controlled substances. Examination of the regulatory status of these drugs in both the UK and the US reveals significant similarities, with only two drugs having different classifications between the two countries. Specifically, while clenbuterol is not considered a controlled substance in the US, it falls under controlled status in the UK. Conversely, butorphanol is not classified as a controlled substance in the UK. Notably, only 13% (2 out of 15) of drugs approved strictly for animal use (carfentanil and pentobarbital) are classified as controlled drugs in both countries.

## 4 DISCUSSION

The primary objective of this systematic review was to delve into the spectrum of veterinary medications prone to misuse or capable of fostering drug-seeking behaviour and dependence in humans while also exploring the motivations behind individuals resorting to substances intended for animal use. To our knowledge, this marks the first systematic literature review analysing the harms associated with veterinary drug misuse in humans.

Of all drugs identified as misused by humans, over half (57% (n=15/28)) are exclusively approved for animal use. Through a comprehensive literature review, we identified 28 distinct veterinary medications being misused by humans. Among these, 15 were solely approved for animal use, while the remaining 13 held approval for both species. Despite certain drugs being approved for both humans and animals, distinct dosages are mandated for each species due to variable biochemical and functional systems, thereby altering the pharmacokinetics of different drugs [26]. Drug metabolism, a crucial aspect of pharmacokinetics, is influenced by the variation in expression and activity of drug-metabolising enzymes between humans and animals, thereby necessitating tailored doses for different species.

Among the 28 drugs identified, their primary effects are attributed to analgesic and sedative properties, indicating potential for misuse. The main drug classes identified in the literature include α-2- and β-2-adrenergic receptor agonists (n=4 drugs), GABAergic receptor modulators (n=4 drugs), opioid receptor agonists (n=3 drugs), NSAIDs (n=3 drugs), and NMDA receptor antagonists (n=2 drugs). Literature findings reveal that veterinary drugs are primarily obtained by individuals working in veterinary settings or those with easy access to the drugs [27-31], as well as through the practices of “vet shopping” and malingering by using animals as proxies [16, 32]. Parenteral injection emerged as the primary route of administration for veterinary drugs, followed by oral ingestion and inhalation by humans. Only 32% of identified veterinary drugs fall under the category of controlled substances (in the UK), with the remaining 68% not subject to the stringent regulations, monitoring, and legal restrictions applied to the prescribing and supply of controlled drugs. The absence of such strict oversight may contribute to increased accessibility and growing misuse of these non-controlled drugs.

The α-2-adrenergic agonists, particularly xylazine, have emerged as a potential contributor to increasing drug-related deaths globally. Xylazine, known for its central nervous system (CNS) depressant effects, is commonly used for sedation, muscle relaxation, analgesia, and anaesthesia in veterinary practice [33]. However, its misuse, often in conjunction with opioids, has potentially led to a surge in fatalities, drawing attention to its alarming presence as an adulterant in illicit drug markets. Studies have documented a sharp increase in xylazine-related deaths by 276% in the US, particularly in combination with illicitly manufactured fentanyl (IMF), indicating a concerning trend in substance misuse [34, 35]. The co-consumption of xylazine and opioids can lead to synergistic effects, exacerbating CNS depression and increasing the risk of fatalities [36]. Recent data underscore the growing prevalence of xylazine in drug-related fatalities, prompting public safety alerts in several countries [37]. Notably, xylazine-associated deaths have been reported in the UK and Europe, indicating its infiltration into the European illicit drug supply [38, 39]. A study found that stimulants were present in 53% of xylazine-positive cases, cannabinoids in 30%, and benzodiazepines in 26% [40]. Other drugs detected in xylazine-related death cases include morphine, cocaine, paracetamol, pregabalin, THC, diazepam, methadone, alcohol, and heroin [39, 41]. Other drugs identified in xylazine-positive syringes included protonitazene, metonitazene, isotonitazene, and carfentanil [42, 43]. The new mixtures of novel benzodiazepines and opioids, with xylazine, have been reported in Estonia [44] and have the potential to seriously impact public health [45]. The mixture of heroin, cocaine, and xylazine, known as ‘speedball’, was found to be obtainable for as little as $8 [46].

Xylazine misuse encompasses various scenarios, including recreational use, adulteration of other drugs, drug-facilitated crimes, and intentional poisoning [47-50]. Using xylazine *via* the intravenous (IV) route was identified as a common factor in 29% of fatalities in one study [51], although subcutaneous, intramuscular, and inhalation methods were also identified [50, 51]. Its combination with opioids, termed “tranq dope”, has been reported to enhance the euphoric effects of fentanyl and prolong its duration of action [52, 53]. Moreover, physical dependence on xylazine, coupled with withdrawal symptoms, has been observed, further complicating its misuse dynamics [54]. Synergistic effects of the combination of opioids with xylazine have been reported to enhance sedation and analgesia in the veterinary setting [55], where greater sedation is observed using the combination than the α-2-agonist alone. Known for inducing painful ulcers, xylazine misuse has been fuelled by its ability to alleviate pain from injection site ulcers it causes, creating a negative cycle. Research shows that α-2 adrenergic agonists, like xylazine, can partially block withdrawal symptoms in opioid users. This suggests individuals may combine xylazine with opioids to manage withdrawal discomfort. Similarly, clonidine, another α-2 agonist, is used to treat withdrawal from various substances by modulating noradrenergic activity. This inhibition of norepinephrine release may explain why xylazine is misused with other drugs. The route of administration for xylazine primarily involves parenteral injection, with males being disproportionately affected by xylazine-related overdoses and fatalities [30, 56, 57]. Despite the increasing prevalence of xylazine in drug markets, there is still limited data on the effects of the drug on the body [58], indicating more research is crucial to further understand the toxic effects in humans.

Medetomidine and dexmedetomidine, α-2-adrenergic agonists primarily used for sedation and analgesia in dogs, have recently emerged as substances of misuse. While dexmedetomidine is approved for both human and animal use, medetomidine is restricted to veterinary use. A toxic adulterant alert in December 2023 [59] identified medetomidine in drug samples containing fentanyl, xylazine, heroin, and cocaine, raising concerns due to its potency and selectivity as an agonist. While xylazine was previously the primary drug in this class associated with diversion and abuse, recent misuse of medetomidine and dexmedetomidine has been observed. Like xylazine, these drugs diminish opioid withdrawal symptoms, potentially explaining their misuse alongside opioids. Additionally, α-2-adrenergic receptors, targeted by these drugs, play a role in modulating symptoms of nicotine and alcohol withdrawal syndromes. Notably, there are no further documented instances of medetomidine or dexmedetomidine misuse in humans beyond the mentioned alert.

Clenbuterol, a β-2-adrenergic receptor agonist, activates adenylyl cyclase and, thus, protein kinase A (PKA) to promote the relaxation of smooth muscles [60]. It is primarily used as a bronchodilator in horses [61] and asthma treatment in humans [62] and has seen a surge in misuse, particularly in the context of weight loss and bodybuilding. Despite its exclusive veterinary approval in the US, clenbuterol has become prevalent in illegal markets and is marketed as a weight loss supplement. The dosages consumed by athletes often far exceed therapeutic levels, reaching up to 200mg daily, posing significant health risks [63]. The addictive potential of clenbuterol misuse stems from its ability to activate the brain's reward system, leading to dopamine release and habit formation [64]. Moreover, the physical effects associated with bodybuilding, such as enhanced athletic performance, increased rate of muscle protein deposition, and reduced appetite, contribute to its addictive behaviour [62, 63]. Salbutamol, a similar β-2 agonist, is also misused for performance enhancement, although clenbuterol exhibits a higher abuse potential due to its potency and pharmacokinetic properties [65].

In addition to its misuse in bodybuilding, clenbuterol is increasingly being mixed with opioids and benzodiazepines, posing grave health risks. The concurrent use of clenbuterol with depressants like opioids and benzodiazepines can lead to unpredictable interactions, exacerbating cardiovascular and respiratory complications [61]. Furthermore, co-ingestion with stimulants like cocaine heightens the risk of cardiovascular distress and central nervous system overstimulation [61]. The widespread availability of clenbuterol online has also fuelled its misuse, with reports of increased exposure to poison control centres [66, 67]. The alarming trend of clenbuterol intoxication showed the presence of heroin, cocaine, fentanyl, benzodiazepines, and methadone [61]. Opioids and benzodiazepines depress both cardiovascular and respiratory functions while inducing sedation in the CNS. In contrast, clenbuterol has opposing effects, boosting heart and respiratory rates while triggering anxiety and tremors in the CNS. Such differing effects can result in unpredictable interactions and heightened risks when these substances are used together. Conversely, cocaine shares similar stimulating effects on the cardiovascular and CNS systems with clenbuterol, escalating the chances of heart complications and CNS overstimulation when these substances are co-consumed.

For the NMDA receptor agonists/antagonists, Telazol, a veterinary anaesthetic (licensed for cats and dogs) compound composed of an equal ratio of zolazepam, a benzodiazepine, and tiletamine, an NMDA receptor antagonist, has raised concerns regarding its misuse in humans despite its safe use in veterinary medicine [28]. The potent nature of tiletamine, akin to ketamine, combined with zolazepam's benzodiazepine properties, poses a risk of misuse and dependence [68]. Instances of Telazol misuse, resembling recreational drugs like ketamine and diazepam, underscore its potential for abuse, particularly among those with easy access to veterinary settings [28]. Despite its controlled status in the US, Telazol remains unregulated in the UK, amplifying concerns regarding its public health impact [69]. In 2003, the UK’s Threat Assessment of Serious and Organised Crime raised concern about the rising abuse of ketamine and further stated that its restriction may lead to Telazol being used as a replacement in the future [70]. Most cases involved individuals with easy access to the veterinary drug combination, indicating a heightened risk within veterinary settings. Telazol misuse by a veterinarian to reduce heroin consumption [71] corroborates with research showing that most Telazol abusers also use other psychoactive drugs, often through cross-addiction, wherein users are more likely to misuse drugs with similar anaesthetic/depressant effects that act on the NMDA/GABA receptors [28]. This pattern of polydrug misuse was evident in a case where a patient was found unresponsive, with Telazol, benzodiazepines, and cannabinoids detected in urine analysis [72]. Tiletamine (a component of Telazol) exhibits significantly higher potency than ketamine, and zolazepam (the other component of Telazol) is 5-10 times more potent than diazepam [73]. Tiletamine, an NMDA receptor antagonist, produces rewarding and reinforcing effects, potentially leading to dependence and addiction [74]. Like ketamine, tiletamine induces hallucinogenic, dissociative effects, possibly contributing to its recreational misuse [71]. Furthermore, NMDA receptor antagonists stimulate the mesolimbic dopamine system and directly inhibit dopamine reuptake, highlighting the role of the reward pathway in drug dependence [74, 75]. Exposure to zolazepam also increases dopamine levels by hyperpolarising GABA neurons, leading to dopamine neuron inhibition [76].

Although ketamine is widely known to be a veterinary anaesthetic, its diversion from medical settings is a contributing factor to its recreational use [77], with high rates of prevalence indicating it has gained prominence as a preferred drug among certain demographics [78, 79]. Until 2021, there was inadequate monitoring of ketamine, which restricted the comprehension of its usage and its impact on public health [80], yet ketamine samples have continuously been present in WEDINOS samples from 2016 to 2023 [81-88]. The misuse of ketamine ‘pink cocaine’ has been associated with increased levels of serotonin, dopamine, and norepinephrine [89], possibly driving its misuse as individuals seek mood enhancement and altered states of consciousness fuelled by the heightened activity of these neurotransmitters. Despite its therapeutic potential in pain management and depression treatment, ketamine's recreational misuse remains a significant health concern [90]. The escalating prevalence of ketamine misuse, highlighted by its emergence as a prevalent substance in drug markets, underscores the urgent need for public health interventions [91, 92]. A rise of ‘pink cocaine’ has been reported, involving ketamine being mixed with other synthetic drugs like MDMA [93], reflecting a rise in consumer interest. The poly-drug misuse of ketamine, particularly in combination with stimulants like cocaine and MDMA, poses grave risks, including cardiovascular complications and serotonin syndrome [94]. Ketamine's pharmacokinetic characteristics include a broad hepatic CYP P-450 induction, which may potentiate the toxicity of other drugs in the hepatobiliary system by increasing the production of harmful metabolites [95]. Despite its therapeutic benefits, ketamine's accessibility, low cost, and potent dissociative effects contribute to its widespread misuse [96].

Opioid agonists identified include carfentanil, tramadol, and butorphanol. Despite its exclusive approval for veterinary use, carfentanil has emerged as a prevalent opioid misused by humans, often disguised as heroin in illicit drug markets [97]. Its potency, estimated to be thousands of times greater than morphine, poses severe health risks, contributing to numerous deaths and poisonings worldwide [98]. Carfentanil's increasing presence in illicit drug markets [99], combined with its potency, makes it particularly dangerous, with users often unaware of its inclusion in street drugs [100]. The year 2017 saw a high increase in prevalence, with 755 seizures reported by seven different countries [101]. The mixture of carfentanil with other substances like cocaine exacerbates these risks, leading to unintended side effects and fatalities [102]. The lack of data on its abuse liability and dependence potential underscores the urgent need for further research and public health interventions [103].

Tramadol, a controlled substance approved for both human and animal use, is susceptible to misuse, particularly due to its accessibility through veterinary prescriptions [6]. Its relatively low cost compared to other opioids and its dual action as an opioid agonist and serotonin-norepinephrine reuptake inhibitor (SNRI) contribute to its abuse potential [32, 104]. Tramadol's unique pharmacological profile results in distinctive withdrawal symptoms and an increased risk of dependence [104, 105]. Despite its partial agonist and antagonist activity, making dependence less likely than with traditional opioids, butorphanol misuse has been documented, often through deceptive means such as malingering by animal proxy [16, 106]. In contrast to other opioids abused by humans, butorphanol demonstrates partial agonist and antagonist activity [106], potentially resulting in a reduced likelihood of dependence compared to opioids like morphine. Limited information exists on butorphanol misuse, highlighting the need for further research and surveillance in veterinary settings.

GABAergic receptor modulators/positive allosteric modulators identified include diazepam, clorazepate, phenobarbital, and pentobarbital. While not commonly discussed in the context of misuse, diazepam stands out as the most prescribed benzodiazepine in veterinary settings [6]. Its accessibility in veterinary medicine raises concerns about potential misuse by pet owners, given its addictive properties and associated withdrawal symptoms. Instances of “vet shopping” and malingering by animal proxy have been documented, illustrating the acquisition of clorazepate from veterinary sources for personal use [16]. As a controlled substance with addictive potential, monitoring its use in veterinary settings is essential, particularly considering the growing concern over benzodiazepine misuse [107]. Used primarily for seizure management in both humans and animals, phenobarbital has been misused, leading to fatal overdoses in some cases [27]. Its accessibility in veterinary medicine poses a risk, especially when individuals with substance use disorders seek to alleviate withdrawal symptoms [26]. Like phenobarbital, pentobarbital misuse has been reported, particularly among individuals associated with veterinary practices [29]. Its potential for habit formation and toxic effects underscores the need for vigilance, especially in professions where access to veterinary medications is common [108]. Recent cases of pentobarbital adulteration in counterfeit fentanyl tablets highlight the potentially lethal consequences of combined drug use [109].

Several veterinary medications, not fitting into previously mentioned categories, have been identified for misuse by humans. Among these are acepromazine, pheniramine, and others.

Acepromazine emerged as a notable focus in five retrieved papers [6, 29, 110-112]. This commonly used phenothiazine tranquiliser is administered to mitigate stress and excitement during various veterinary procedures [113]. Originally approved for treating schizophrenia in humans, acepromazine is now exclusively licensed for veterinary use, although it is not classified as a controlled substance. Its pharmacological profile includes antagonistic effects on dopaminergic and serotonin receptors, as well as antagonism of histamine, muscarinic acetylcholine, and α-1 receptors [110]. A case study detailed a woman who intentionally ingested 950 mg of her dog’s acepromazine, with a medical history notable for depression, anxiety, and hypothyroidism. Despite several reports of acepromazine poisonings, including instances of drug-facilitated sexual assaults and suicides, detection remains challenging due to rapid metabolism [112]. A case where a survey of veterinary practitioners revealed misuse of veterinary acepromazine for stress management [111]. In these cases, acepromazine misuse appeared associated with mental health conditions such as stress, anxiety, and depression, possibly linked to its antagonism of dopamine and serotonin receptors. Two additional suicide cases involving acepromazine were documented [29], both involving female individuals. One case involved a veterinary worker, while the source of acepromazine for the other patient remained unclear. In both instances, acepromazine was implicated in completed suicides, with one dose totalling 2500 mg. To our knowledge, the misuse of its analogue promazine has not been documented and is not known.

Pheniramine, an antihistamine, is approved for use in both humans and animals, primarily targeting allergic conditions. Antihistamines, easily accessible over the counter, rank among the most abused drugs [114]. A study revealed that 14.7% of overdose deaths in the US between 2019 and 2020 involved antihistamines, with opioids implicated in 82.8% of these cases [115]. However, despite this concerning trend, the UK has not analysed antihistamine-related mortalities in over 40 years [116], and reports on pheniramine misuse are scarce. Notably, a high proportion (79.9%) of patients hospitalised due to pheniramine poisoning had a history of drug or alcohol abuse, with 60.5% exhibiting an antihistamine abuse history [117]. Although not a controlled substance, one documented case highlights veterinary grade pheniramine misuse, where a user intravenously mixed 100 mg of heroin with 15 ml of injection pheniramine 4-5 times daily in an attempt to manage sleep issues [118]. Co-administration of pheniramine with opioids like heroin can lead to life-threatening outcomes, given the additive effects of antihistamines with CNS depressants [116]. In this case, the user exhibited signs of heavy pheniramine addiction, experiencing withdrawal symptoms such as insomnia, restlessness, and tremors upon attempts to reduce dosage [118]. Psychological tolerance and physical withdrawal symptoms to pheniramine misuse have been documented [118]. It remains unclear how the veterinary-grade pheniramine was obtained in this case, but a 100 ml bottle labelled “NOT FOR HUMAN USE. FOR ANIMAL TREATMENT ONLY” was reported. Given its source outside traditional pharmacies, it's plausible that this veterinary product was purchased online. A study addressing the illicit veterinary medicine market highlighted the distribution of such medications through illegal online pharmacies, online marketplaces, and social media platforms, posing significant regulatory and enforcement challenges [119].

Stanozolol, an anabolic steroid, holds licenses for use in both human and veterinary medicine and is classified as a Class C controlled substance [120]. It ranks among the most abused anabolic androgenic steroids (AAS), particularly among young adults and professional athletes, who often seek to enhance physical appearance and performance [121]. A case study documented an individual's attempt to procure stanozolol without an accompanying animal from a veterinary facility [16]. While the extent of the individual's dependence on the AAS remains unclear, studies have indicated the potential for dependency due to the self-administration stimulation observed in animal models [122]. Although users do not experience immediate intoxication, dependence on AAS may develop, particularly in individuals grappling with body image disorders like “muscle dysmorphia” [123].

Both levothyroxine and furosemide were found to be utilised inappropriately for weight loss [16, 111]. Levothyroxine, typically prescribed for hypothyroidism, was acquired from a veterinary source by a veterinary worker for off-label use as a weight loss aid. It was apparent that in this instance, the individual engaged in 'vet shopping', obtaining multiple prescriptions for levothyroxine from different veterinary clinics. Similarly, misuse of furosemide, a loop diuretic, was reported by veterinarians to be misused for weight management [111]. Furosemide has been recognised for its misuse in sports due to its ability to induce rapid weight loss [124].

A single case of veterinary amitriptyline misuse was identified. Amitriptyline, a tricyclic antidepressant, is licensed for use in both humans and animals. A detailed incident wherein an anxious pet owner specifically requested amitriptyline for her dog [16]. The prescribed three-week medication supply was depleted within a mere 10 days, prompting suspicion of misuse by the owner. Notably, a study revealed that 25% of amitriptyline users aimed to achieve euphoria [125], highlighting the potential for dependence and abuse. This may be attributed to the drug's synergistic antihistamine and anticholinergic effects [126].

Two articles documented the misuse of the veterinary antibiotic tilmicosin. While this antibiotic serves as a calcium-channel blocker and lacks approval for human use, it has been implicated in suicide cases. Tilmicosin poses a significant risk to certain animal species, including pigs, primates, and horses, due to its cardiotoxicity [62]. However, it is deemed appropriate for treating specific infectious diseases in cattle and sheep. Despite many exposures being accidental, there have been 25 recorded deaths, with 16 suspected suicides [127]. The primary exposure route in all tilmicosin cases was parenteral [29], with intentional misuse in humans attributed to its widespread availability. In 2017, the FDA issued a warning regarding the dangers of tilmicosin, noting its lack of antidote and its toxic effects on the heart [127].

The use of Tanax^®^ has been implicated in suicide cases. Tanax^®^ is a veterinary drug comprising three ingredients: embutramide (a general anaesthetic), mebezonium iodide (a neuromuscular blocking agent), and tetracaine hydrochloride (a local anaesthetic), known to potentially encourage abuse due to its hypnotic effects [128]. Before 2014, eight documented fatalities resulted from the self-administration of mebezonium and embutramide [29]. Notably, 50% of these cases involved individuals with convenient access to euthanasia agents, including veterinarians. Forensic and clinical toxicological analyses revealed embutramide in two cases in 2013 [128]. In the first case, embutramide was detected in the urine of a man who had murdered his ex-wife along with alprazolam. The second case involved a 16-year-old hospitalised for severe symptoms, experiencing recurrent episodes of unconsciousness, bradycardia, and diplopia over several months. While research [128] indicated that this drug combination had not previously been associated with abuse, both cases underscored the need for heightened attention to the misuse of veterinary medications.

Dinoprost and cloprostenol are both classified as veterinary medications with potential hazards for humans. Dinoprost, a synthetic form of prostaglandin F2 alpha, is not approved for human use and is primarily employed for inducing abortion in cattle [62]. However, concerns have been raised regarding its potential misuse for terminating unwanted pregnancies in humans. There are reports of a case of dinoprost misuse for this purpose [111]. In contrast, it was noted that human exposure to dinoprost is typically accidental, often occurring through occupational exposure [62]. Similarly, cloprostenol, another synthetic prostaglandin used in veterinary medicine, is not licensed for human use and shares concerns about potential misuse for inducing abortion.

Interestingly, phenylbutazone emerged as the most frequently misused veterinary medication, constituting 57% of all reported cases involving non-steroidal anti-inflammatory drugs (NSAIDs). While primarily intended for animal use, phenylbutazone is approved for treating ankylosing spondylitis in humans. However, its human usage is associated with gastrointestinal toxicity, renal dysfunction, and aplastic anaemia [111]. Concerningly, instances of phenylbutazone adulterating illicit drugs, particularly those containing heroin, fentanyl, and/or fentanyl derivatives, have been on the rise [129]. This trend is troubling, given that phenylbutazone was largely discontinued for human consumption due to associated fatalities. Since 2016, Pennsylvania alone has reported 116 positive samples containing phenylbutazone as an adulterant [129]. Flunixin, another NSAID, was identified as a medication misused in a study analysing veterinarians' perceptions of the misuse of veterinary medications in humans [111]. While NSAIDs were the most frequently reported class of drugs in this study, flunixin accounted for 24% of these cases. Adverse outcomes associated with flunixin's misuse in humans, including gastrointestinal toxicity and renal dysfunction, were documented [111]. The study highlighted the potential for severe human overdose due to the oral formulations of flunixin used for horses. Similarly, carprofen, another veterinary NSAID, was recognised as being misused by humans in the same study [111]. Although NSAIDs were the most misused drug class identified, carprofen ranked as the third most misused drug within this category (13%). However, no additional reports of flunixin or carprofen misuse were found in the literature. In general, over-the-counter NSAIDs are known to have an increasing potential for misuse [130]. This is because of their availability and overuse.

Levamisole, a veterinary anti-parasitic, has consistently been identified in cocaine seizures since 2002, despite it no longer being approved for human use [131]. A study analysing seized drug samples demonstrated that levamisole was one of the most prevalent adulterants, found in 11.8% of samples [132]. Although the exact reasons for the use of levamisole as an adulterant in cocaine are unclear, several theories suggest its increase in prevalence is associated with the inhibition of dopamine reuptake in the presynaptic terminals of neurones, as well as the potentiation of the nicotinic acetylcholinergic effects, both enhancing the euphoric effects of the cocaine [132]. Given its ongoing presence in illicit drug supplies and its potential health risks, such as neutropenia and agranulocytosis [132], continued monitoring remains crucial in understanding its impact and mitigating associated harms.

Finally, the misuse of a veterinary vitamin supplement containing vitamins A, D, and E to enhance muscle volume served as a primary motivation behind this misuse, with the oily vehicle of the supplement contributing to this effect [133]. Over four months preceding the case presentation, a parenteral application of 150 mL, containing 20,000,000 IU of vitamin A, 5,000,000 IU of vitamin D3, and 6,800 IU of vitamin E per 100 mL vial, was administered. Despite being restricted for veterinary use only, this vitamin combination is becoming increasingly popular in Brazil due to its non-anabolic classification, easy accessibility, and affordability [133]. Although not inherently addictive, users may misuse the supplement due to observable physical changes and may develop psychological dependence to achieve fitness goals. Several other studies also document the misuse of the veterinary ADE supplement for bodybuilding purposes [134, 135]. However, all reported cases are from South America, and it remains unclear whether similar misuse occurs in the UK.

The findings presented shed light on a concerning trend of increasing misuse of veterinary medications, reflecting a complex interplay of factors driving this phenomenon. While most data primarily focus on misuse in the US and UK, there are significant reports of carfentanil misuse across Northern Europe [136]. Additionally, the detection of xylazine has extended to Estonia, Latvia, and France [137], demonstrating that this issue is widespread. The accessibility and affordability of these drugs, coupled with lax prescribing oversight, have rendered them attractive to a diverse range of users for various purposes, from recreational use to self-medication and even illicit drug adulteration. However, the underreporting of such instances highlights a significant gap in our understanding of the scope and implications of veterinary drug misuse. Furthermore, the diverse motivations behind this misuse, including recreational, therapeutic, and criminal intents, underscore the need for multifaceted interventions to address this issue effectively. Strengthening monitoring protocols within the veterinary industry and enhancing public awareness and education are crucial steps towards mitigating the risks associated with veterinary drug misuse. Additionally, healthcare professionals must remain vigilant to the unique challenges posed by poly-substance use involving veterinary medications, necessitating the development of targeted treatment and intervention strategies. Ultimately, concerted efforts across multiple sectors are essential to address this emerging public health concern and safeguard both human and animal welfare.

## 5 LIMITATIONS

As the misuse of veterinary medicines in humans is an emerging phenomenon, a limitation for this study includes the lack of substantial evidence in this subject area. Although a risk of bias assessment was conducted, there were a large amount of case report studies. These studies carry a higher risk of bias and may affect the interpretation of the findings. Further research is needed to gain a better understanding of this developing trend.

## CONCLUSION

This comprehensive literature review aimed at evaluating the prevalence and motivations underlying the misuse of veterinary medications reveals a troubling trend. Veterinary drugs are increasingly appealing to drug users due to their affordability and ease of access, stemming from less rigorous prescribing oversight. However, despite this surge in usage, instances of veterinary medication misuse remain largely underreported, with scant data available for research. The review revealed various rationales driving this misuse, ranging from recreational use to pain relief, self-medication, suicide, drug-facilitated crimes, pregnancy termination, bodybuilding, and weight loss. Of particular concern is the frequent use of veterinary drugs as adulterants in illicit drug samples, often unclaimed to consumers, leading to unintended exposures and potential health hazards.

There exists an urgent need for veterinary professionals to bolster monitoring protocols for their products, aiming to curtail overdose incidents among staff and associated personnel while also ensuring that animal owners procure these drugs for legitimate purposes. Concurrently, healthcare practitioners must exercise heightened vigilance regarding the diverse effects that may manifest in emergency room scenarios due to poly-substance use, exacerbated by the lack of necessary antidotes for many veterinary products. To effectively address these challenges, a multi-pronged approach is imperative. This includes bolstering public awareness and education efforts to elucidate the risks associated with veterinary medications. Furthermore, stricter regulatory measures are warranted alongside the development of more robust treatment and intervention strategies to mitigate the burgeoning misuse of these medications.

## Figures and Tables

**Fig. (1):**
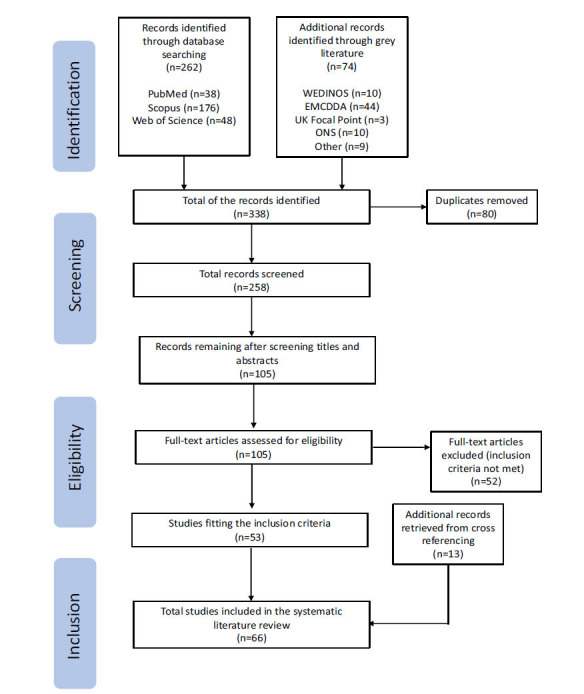
PRISMA flow diagram of included studies assessing the effects of veterinary medication use by humans on their health (*Welsh Emerging Drugs and Identification of Novel Substances (WEDINOS), European Monitoring Centre for Drugs and Drug Addiction (EMCDDA), Office for National Statistics (ONS)).

**Table 1:** Drugs identified through systematic literature search and their potential reasons for their misuse in humans.

**Drug Class**	**Name of Drug**	**Reason for Misuse in Humans**
Adrenergic Receptor Agonists	Xylazine	Sedation/Analgesia
	Medetomidine	Sedation/Analgesia
	Dexmedetomidine	Sedation/Analgesia
	Clenbuterol	Performance Enhancement
NDMA Antagonists	Ketamine	Analgesia/Dissociation/Sedation
	Telazol (Zolazepam/Tiletamine)	Anaesthesia/Sedation/Sedation
Opioid Receptor Agonists	Carfentanil	Analgesia/Euphoria
	Tramadol	Analgesia/Sedation/Euphoria
	Butorphanol	Analgesia/Sedation/Euphoria
GABAergic Receptor Modulators	Diazepam	Sedation/Muscle Relaxation
	Clorazepate	Sedation/Muscle Relaxation
	Pentobarbital	Suicidal Indications/Sedation
	Phenobarbital	Sedation/Anticonvulsant/Hypnotic Effects
Other Drugs	Acepromazine (Phenothiazines)	Sedation/Muscle Relaxation
	Levamisole (Anthelminthic)	Bulking agent
	Pheniramine (Antihistamine)	Sedation
	Stanozolol (Anabolic Steroid)	Performance Enhancement
	Levothyroxine (Thyroid Hormone)	Weight loss Supplement
	Furosemide (Loop Diuretic)	Weight loss Supplement
	Amitriptyline (Tricyclic Antidepressant)	Antidepressant Properties
	Tilmicosin (Macrolide Antibiotic)	Suicidal Indications
	Embutramide/Mebezonium (Euthanasia Compound)	Suicidal Indications
	Dinoprost (Prostaglandin)	Abortion
	Cloprostenol (Prostaglandin)	Abortion
	Phenylbutazone (NSAID)	Analgesia/Anti-Inflammatory
	Flunixin (NSAID)	Analgesia/Anti-Inflammatory
	Carprofen (NSAID)	Analgesia/Anti-Inflammatory
	Vitamin ADE Compound	Performance Enhancement

**Table 2:** The veterinary drugs identified from the literature review, their licensed usage, and legal classification in both the UK and the US.

**Drug Name**	**Approved Usage (Humans or Animals)**	**Status: UK**	**Status: FDA**
Xylazine	Animals	Not Controlled	Not Controlled
Medetomidine	Animals	Not Controlled	Not Controlled
Dexmedetomidine	Both	Not Controlled	Not Controlled
Clenbuterol	Both	Class C, Schedule 4	Not Controlled
Ketamine	Both	Class B, Schedule 2	Schedule III
Telazol (Zolazepam/Tiletamine)	Animals	Not Controlled	Not Controlled
Carfentanil	Animals	Class A	Schedule II
Tramadol	Both	Class C, Schedule 3	Schedule IV
Butorphanol	Both	Not Controlled	Schedule IV
Diazepam	Both	Class C, Schedule 4	Schedule IV
Clorazepate	Both	Class C, Schedule 4	Schedule IV
Pentobarbital	Animals	Class B, Schedule 3	Schedule II
Phenobarbital	Both	Class B, Schedule 3	Schedule IV
Acepromazine (Phenothiazines)	Animals	Not Controlled	Not Controlled
Levamisole (Anthelminthic)	Animals	Not Controlled	Not Controlled
Pheniramine (Antihistamine)	Both	Not Controlled	Not Controlled
Stanozolol (Anabolic Steroid)	Both	Class C, Schedule 4	Schedule III
Levothyroxine (Thyroid Hormone)	Both	Not Controlled	Not Controlled
Furosemide (Loop Diuretic)	Both	Not Controlled	Not Controlled
Amitriptyline (Tricyclic Antidepressant)	Both	Not Controlled	Not Controlled
Tilmicosin (Macrolide Antibiotic)	Animals	Not Controlled	Not Controlled
Embutramide/Mebezonium (Euthanasia Compound)	Animals	Not Controlled	Not Controlled
Dinoprost (Prostaglandin)	Animals	Not Controlled	Not Controlled
Cloprostenol (Prostaglandin)	Animals	Not Controlled	Not Controlled
Phenylbutazone (NSAID)	Animals	Not Controlled	Not Controlled
Flunixin (NSAID)	Animals	Not Controlled	Not Controlled
Carprofen (NSAID)	Animals	Not Controlled	Not Controlled
Vitamin ADE Supplement	Animals	Not Controlled	Not Controlled

## LIST OF ABBREVIATIONS

CNS: Central Nervous System
IMF: Illicitly Manufactured Fentanyl
NSAIDs: Non-steroidal Anti-inflammatory Drugs
PKA: Protein Kinase A
SNRI: Serotonin-norepinephrine Reuptake Inhibitor
VPMs: Veterinary Prescription Medications

## SUPPLEMENTARY MATERIAL

PRISMA checklist is available as supplementary material on the publisher’s website along with the published article.
