# Supplementary material for: Exploring the Confluence of Animal Medicine and its Implications for Human Health: A Systematic Literature Review
Source: Curr Neuropharmacol. 2024 Sep 16;23(7):847–61. doi: 10.2174/011570159X333443240822115028 (PMC12172238; doi:10.2174/011570159X333443240822115028)
Supplement: Supplementary file 1 [file CN-23-7-847_SD1.pdf]

Supplementary Material

Exploring the Confluence of Animal Medicine and its Implications for Human Health: A Systematic Literature Review

Josie Dunn[1], Fabrizio Schifano[2], Ed Dudley[1] and Amira Guirguis[1,*]

[1]Medical School, The Grove, Swansea University, Swansea, SA2 8PP, UK; [2]Psychopharmacology, Drug Misuse and Novel Psychoactive Substances Research Unit, School of Life and Medical Sciences, University of Hertfordshire, Hatfield, AL10 9AB, UK

1875-6190/25 $65.00+.00 **© 2025 Bentham Science Publishers**

Table S1. All articles included within the systematic literature review.

Type of Publication	Main Findings	Specifics - illicit Indication	Dose	Route	Source	Reference Number
Case Study https://www.ncbi.nlm.nih.gov/pmc/articles/PMC4371025/	950mg of a woman's dog's acepromazine was ingested intentionally, resulting in central nervous system and respiratory depression. Her past medical history included depression, anxiety and hypothyroidism.	Potential self-medication to treat anxiety/depression	950mg	Oral	Pet's own prescription	110
Case Study https://www.ncbi.nlm.nih.gov/pmc/articles/PMC9249150/	A 29-year-old male reported misuse of injected 100mg heroin mixed with 15ml veterinary-use pheniramine maleate, 4-5 times a day. Misuse started due to sleep problems and decreasing the dose lead to insomnia, restlessness, and tremulousness. The likelihood for addiction potential is due to stimulation of dopamine. Case report concluded pheniramine has a dependence potential.	Initially misused for sleep problems	100mg heroin with 15ml pheniramine	Parenteral injection	Online Source	118
Case Study https://www.ncbi.nlm.nih.gov/pmc/articles/PMC7473675/	Myocardial injury is one of the life-threatening complications due to the misuse of clenbuterol. Although used in veterinary medicine, it's misuse has been increasing due to the illegal marketing as a weight loss supplement. There is no reported antidote for clenbuterol misuse.	Anabolic effects for bodybuilding	40mg (dosing frequency unknown. Most commonly dosing regimen in athletes if up to 200mg, 1-3 times daily	Oral	N/A	63
Case Study https://pubmed.ncbi.nlm.nih.gov/18713522/	Discussed 12 clenbuterol cases of intoxication. Heroin was present in 8/12 cases with the remaining 4 cases indicating a history of heroin misuse due to the presence of morphine. Multi-drug use was popular with fentanyl present in 3 cases, cocaine in 4, ethanol and benzodiazepines in 2, and methadone present in 1 case.	Anabolic effects for bodybuilding	Case 1 - 76 ng/mL (Blood)<br>Case 2 - Present (Urine), Trace (Blood)<br>Case 3 - 7.6ng/mL (Blood)<br>Case 4 - Present (Urine), Trace (Blood)<br>Case 5 - Present (Urine), ND (Blood)<br>Case 6 - 10ng/mL (Blood)<br>Case 7 - 5.5ng/mL (decomposition fluid), 12ng/g (Spleen)<br>Case 8 - Present (Urine), ND (Blood)<br>Case 9 - Present (Urine), Trace (Blood)<br>Case 10 - Present (Urine), ND (Blood)<br>Case 11 - Present (Urine), 6.3ng/mL (Blood)<br>Case 12 - Present (Urine), 20ng/mL (Blood)	N/A	N/A	61
Case Study https://onlinelibrary.wiley.com/doi/10.1111/1556-4029.13010	1/3 of Tanax's components, Embutramide, was identified in the urine of a man who murdered his ex-wife, along with alprazolam. The second case study reported a 16-year-old who was hospitalised, where embutramide, drotaverine (antispasmodic) and alprazolam was found. This patient suffered with severe symptoms and was hospitalised 4 more times in the following 4 months due to	Suicide attempt (case 1) & general drug misuse (case 2)	2.36 µg/mL (in urine (case 1)) & 2.83µg/mL (in urine (case 2))	Case 1 - N/A<br>Case 2 - Inhaled	Case 1 - User was a veterinarian so had own access<br>Case 2 - N/A	128

Type of Publication	Main Findings	Specifics - illicit Indication	Dose	Route	Source	Reference Number
	the same symptoms, being periods of unconsciousness, bradycardia and diplopia.					
Case Study https://pubmed.ncbi.nlm.nih.gov/29319776/	Veterinary concentrations of vitamin A, D and E were misused and injected into patient's arms twice a month. This veterinary vitamin combination is popular in Brazil due to its availability and low cost, and due to not being classed as an anabolic steroid.	Muscle swelling for body building	150mL of vitamin ADE (20,000,000 IU Vitamin A, 5,000,000 IU Vitamin D3, 6,800 Vitamin E per 100mL vial) in the previous 4 months	Parenteral injection	N/A	135
Case Study https://www.sciencedirect.com/science/article/pii/S0735675709000102?via%3Dihub	A 35-year-old veterinarian was hospitalised with movement disorder due to the misuse of Zoletil (Telazol) - a fixed ratio combination of zolazepam (tranquiliser) and tiletamine (anaesthetic). The accessibility of scheduled drugs and health care professionals was highlighted in this case.	To reduce the amount of heroin misused	N/A	N/A	User was a veterinarian so had own access	71
Case Study file:///C:/Users/2009986/Downloads/Phenobarbitaltoxicityfromahighlyconcentrated-edveterinaryformulation-reviewandcasereport%20(1).pdf	Case of intoxication of a high concentration of veterinary acquired phenobarbital, complicated by ethanol abuse. The co-ingestion caused significant central nervous system depressants.	Patient had a history of substance misuse	124mcg/mL initial serum concentration (consumption amount unknown)	N/A	User was an assistant horse trainer with access to equine phenobarbital	27
Case Study https://academic.oup.com/jat/article/25/4/245/779255?login=true	The dose of xylazine used for animals ranges from 0.5-5.0mg/kg. A 27-year-old farmer attempted suicide with an ~75mL 2% aqueous solution xylazine by intramuscular injection.	Suicide attempt	75mL 2% aqueous solution	Intramuscular	User was a farmer	31
Case Study https://pubmed.ncbi.nlm.nih.gov/12539907/	5 cases of malingering my animal proxy were reported by veterinarians. Case 1 involves a dog noise phobia case in order to receive clorazepate (benzodiazepine) for the owner's use. Case 2 includes a false reporting of malnutrition in a dog to obtain stanozolol. In case 3, the client was seeing multiple veterinary clinics to misuse levothyroxine for weight loss. Case 4 involved falsely reporting a dog's cough to receive opioids. Case 5 involved amitriptyline for the owner to misuse as an antidepressant.	Case 1 - Benzodiazepine (Clorazepate) misuse<br>Case 2 - Anabolic steroid use for body building<br>Case 3 - Levothyroxine misuse for weight loss<br>Case 4 - Opioid (Tobuterol) misuse<br>Case 5 - Self-medication for anxiety using amitriptyline	Case 1 - 7.5mg<br>Case 2 - N/A<br>Case 3 - N/A<br>Case 4 - 7- to 10-day 5mg<br>Case 5 - 30mg every 12 hours for 21 days	Case 1 - Oral<br>Case 2 - N/A<br>Case 3 - N/A<br>Case 4 - Oral<br>Case 5 - Oral	Prescribed from veterinary clinic	16
Case Study https://pubmed.ncbi.nlm.nih.gov/29098704/	Describes three cases where xylazine was used in human poisoning events with criminal intent via drink spiking. This report suggests xylazine should be classified as a controlled drug.	Intentional poisoning with criminal intent	Case 1 - N/A<br>Case 2 - 0.294 µg/mL (urine) & 0.057 µg/mL (serum)<br>Case 3 - 0.533 µg/mL (urine)	Oral ingestion	N/A	48
Case Study https://pubmed.ncbi.nlm.nih.gov/11527235	A 30-year-old zoo employee, found unresponsive, tested positive for benzodiazepines and cannabinoids and revealed a history of	Patient revealed history of Telazol	N/A	Parenteral injection	User was a veterinary worker	72

Type of Publication	Main Findings	Specifics - illicit Indication	Dose	Route	Source	Reference Number
/	Telazol misuse.	recreational misuse				
Case Study https://pubmed.ncbi.nlm.nih.gov/10872580/	A 22-year-old male was found dead with 28 needle marks where it was suspected illicit drugs were used. Upon analysis, tiletamine and zolazepam were identified. This drug combination is common in veterinary medicine as an anaesthetic.	N/A	Exact doses unknown Concentration in blood = 0.85mg/L (tiletamine) & 3.3mg/L (zolazepam) Concentration in tissue injection site = 25.2mg/L (tiletamine) & 23.3mg/L (zolazepam)	Parenteral injection	N/A	73
Case Study https://pubmed.ncbi.nlm.nih.gov/12670006/	A case study in which xylazine was detected on its own in a suicide by hanging.	Suicide due to history of depression	The detected xylazine concentrations were as follows: 2.3 mg/L in heart blood, 2.9 mg/L in peripheral blood, 6.3 mg/L in bile, 0.01 mg/L in urine, 6.1 mg/kg in the liver, and 7.8 mg/kg in the kidney.	Parenteral injection	User was a veterinary worker	49
Case Study https://pubmed.ncbi.nlm.nih.gov/37236142/	The first drug-related death in the UK/Europe associated with Xylazine was reported to the National Programme on Substance Abuse Deaths (NPSAD) on the 31/12/22. Other drugs present in urine/blood samples of the deceased included cocaine, fentanyl, morphine, paracetamol, pregabalin, THC, diazepam, methadone and alcohol.	Illicit drug misuse - also found cocaine, fentanyl, diazepam and alcohol in tissue	Exact dose unknown Blood concentration of xylazine = 38ng/ml and urine = 135ng/ml	N/A	N/A	38
Government Article https://www.gov.uk/government/publications/controlled-drugs-list--2/list-of-most-commonly-encountered-drugs-currently-controlled-under-the-misuse-of-drugs-legislation	A compilation of the frequently encountered drugs currently regulated by the misuse of drugs legislation, indicating their classifications under both the Misuse of Drugs Act 1971 and the Misuse of Drugs Regulations 2001.	N/A	N/A	N/A	N/A	120
Government Report https://www.gov.uk/government/publications/united-kingdom-drug-situation-focal-point-annual-report/uk-drug-situation-2019-summary	The current rate of ketamine use among adults in England and Wales is the highest ever recorded, reaching 0.8%.	N/A	N/A	N/A	N/A	91
Informative poster https://www.avma.org/sites/default/files/2019-11/Opioids_Vet-Shopping-Drug-Diversion_Guide-for-Veterinarians_flyer.pdf	Describes behaviour associated with 'vet shoppers' and ways to minimise drug diversion in a veterinary setting.	N/A	N/A	N/A	N/A	10
Journal Article https://pubmed.ncbi.nlm.nih.gov/27341080/	Reviewed 7 cases of human exposure to the veterinary tiletamine-zolazepam combination. In 6/7 cases, administration was intentional and the use of the drug combination in 5/7 cases was for recreational purposes. It was shown that human misuse of veterinary medications is more prevalent than previously thought. The majority of people who	Case 1 - Recreational use<br>Case 2 - Substitute for heroin<br>Case 3 - To get high<br>Case 4 - N/A<br>Case 5 - To get high<br>Case 6 - Suicidal	Case 1 - 200mg-100mg tiletamine, 100mg zolazepam<br>Case 2 - N/A<br>Case 3 - 500mg<br>Case 4 - 875mg tiletamine, 875mg zolazepam<br>Case 5 - 1125mg tiletamine, 1125mg zolazepam (over 9 days)	Case 1 - Injection<br>Case 2 - N/A<br>Case 3 - Ingestion<br>Case 4 - Injection<br>Case 5 - Injection<br>Case 6 - Injection<br>Case 7 - Injection	2/7 patients were veterinarians, 1/7 works in a veterinarian's office, 1/7 is a zoo employee	28

Type of Publication	Main Findings	Specifics - illicit Indication	Dose	Route	Source	Reference Number
	misuse the TZ combo also use or abuse other psychoactive substances.	purposes<br>Case 7 - Recreational use	Case 6 - N/A<br>Case 7 - N/A			
Journal Article https://pubmed.ncbi.nlm.nih.gov/37644817/	Acepromazine poisonings have been reported, including suicide reports and drug-facilitated sexual assaults, however it is difficult to detect due to rapid metabolism.	N/A	N/A	N/A	N/A	112
Journal Article https://medic.upm.edu.my/upload/dokumen/2022071815362726_MJMHS_1600.pdf	Xylazine can be misused in several ways including as a recreational drug, an adulterant, in drug-facilitated crime/sexual assault and as a source of accidental and intended poisoning.	Recreational drug, adulterant, drug facilitated crime and sexual assault, doping agent in animal sport	N/A	Inhaled/snorted/ injected	N/A	47
Journal Article https://pubmed.ncbi.nlm.nih.gov/33403403/	Currently, there is insufficient information regarding veterinary prescription drug misuse to estimate the severity. 398 veterinarians reported in a study that they suspected 23% of pet owners misuse animal drugs on themselves. A different study found that 13% of veterinarians were conscious of an animal owner that injured their pet to gain opioids, and 12% were aware of staff opioid misuse. Opioid prescribing is increasing in the veterinary setting.	N/A	N/A	N/A	Veterinary setting	6
Journal Article https://jamanetwork.com/journals/jama/fullarticle/2805530	Xylazine was found in every street opioid sample tested by the Philadelphia Department of Public Health in January 2023. The FDA have issued an import alert, restricting unlawful importation of xylazine, in February 2023. In April 2023, the White House Office of National Drug Control Policy declared xylazine mixed with fentanyl as an "emerging threat to the United States".	N/A	N/A	Injection	Illicit drug supply	53
Journal Article https://pubmed.ncbi.nlm.nih.gov/20045604/	There is a need for increased awareness of the potential hazards of veterinary medications in humans. The veterinary products with significant health hazards to humans are carfentanil, clenbuterol, ketamine, tilmicosin, testosterone/estradiol, dinoprost and cloprostenol.	N/A	N/A	N/A	N/A	62
Journal Article https://pubmed.ncbi.nlm.nih.gov/25404261/	Examined which veterinary compounds are misused in human suicide. The drugs found were veterinary-grade pentobarbital, xylazine, tilmicosin (antibiotic), acepromazine and euthanasia preparations (mebezonium and embutramide).	Pentobarbital - Suicide<br>Acepromazine - Suicide	Lethal blood concentration of 2mg/L of pentobarbital reported, 2500mg acepromazine, 21mg/kg tilmicosin	Parenteral/oral consumption	50% of cases involved either veterinarian/those who had easy access due to their employment. Reports of people with no association	29

Type of Publication	Main Findings	Specifics - illicit Indication	Dose	Route	Source	Reference Number
					with veterinary medicine being able to successfully buy veterinary-grade pentobarbital.	
Journal Article https://pubmed.ncbi.nlm.nih.gov/29733092/	In America, veterinarians are a unique source of prescription opioid analgesics as many states do not need to report their prescribing of them. There are no limits on the amounts of opioids veterinarians can prescribe, influencing diversion/misuse. 75% of a sample (of US veterinarians) were aware of working with someone with a substance abuse problem.	N/A	N/A	N/A	Veterinary setting	32
Journal Article https://pubmed.ncbi.nlm.nih.gov/12135152/	Analgesic, anti-inflammatory, anti-arthritis, systemic antibiotics and topical corticosteroids were the most frequently reported veterinary drugs misused. Veterinarians stated the most likely reason for veterinary drug misuse include lower cost, convenient availability and the belief that veterinary medications are stronger than comparable human medications.	Low cost and the belief that veterinary medications are stronger than comparable human medications	N/A	N/A	People involved in animal sport, those who work in healthcare	111
Journal Article https://pubmed.ncbi.nlm.nih.gov/21481268/	There is a notable gap in the understanding of how xylazine was diverted into the illicit drug market, the specific context of its use, and the chronic health implications associated with its consumption. It was common for consumers in Puerto Rico to be able to control the ratio of heroin:xylazine themselves, as it was usually sold not mixed. 'Speedball' was a mix of heroin, xylazine and cocaine and when sold, each substance were kept separate so the user could tailor to their own liking. Skin ulcers, due to xylazine, promoted further xylazine use to help manage the pain.	N/A	N/A	Parenteral injection	N/A	54
Journal Article https://www.sciencedirect.com/science/article/pii/S0736467916303547	There were 76 cases of xylazine exposures reported to Texas poison centres between 2000-2014. 93% of patients were over the age of 20 and 54% were male. Injection accounted for 51% of exposures and ingestion for 28%. 64% of exposures were unintentional and 32% were intentional. Drowsiness/lethargy (47%), bradycardia (20%), hypotension (11%), hypertension (9%), puncture/wound (8%) and slurred speech (8%) were the most common clinical effects.	31.6% of exposures were intentional, of which 15.8% were suspected suicide attempt, 13.2% were drug abuse	N/A	Parenteral injection (51.3%), Ingestion (15.8%), Dermal Route (14.5%), Ocular Route (2.6%), Inhalation (2.6%).	N/A	57

Type of Publication	Main Findings	Specifics - illicit Indication	Dose	Route	Source	Reference Number
Journal Article https://pubmed.ncbi.nlm.nih.gov/24769343/	From 1966 to 2013, 43 cases of intoxication were reported, of which 51% resulted in fatalities. Of the 22 fatal instances, 17 had xylazine usage as a contributing factor. Males made about 60% of the intoxication cases. In 82% of cases, xylazine deaths were accidental, whereas 9% were suicide-related. Xylazine was employed in 17/18 unintentional occurrences as an adulterant. Parenteral (intramuscular, subcutaneous, and intravenous) administration was the primary mode of delivery. 33% of intoxications were individuals that had easy access to the drug, including veterinarians (and assistants), farmers and horse trainers.	Horse doping agent, a drug of abuse, for attempted sexual assault, as a source of accidental or intended poisonings	Doses to produce toxicity and fatality vary from 40 to 2400ng	Inhaled, intramuscular, intravenous, ocular exposure, oral administration, subcutaneous, self-administration	Individuals who had easy access (veterinarians /farmers/horse trainers)	30
Journal Article https://pubmed.ncbi.nlm.nih.gov/37009344/	Of the 59 documented occurrences of xylazine intoxication, 21 had fatal results; of these, 17 included the combination of xylazine and other substances. 1,200mg was the average fatal dose, 525mg was the average dose in non-fatal cases.	Drug abuse	525mg = non-fatal average dose<br>1,200 mg = fatal average dose<br>Doses ranged from 40mg-4300mg	Intravenous, subcutaneous, intramuscular, inhalation	N/A	51
Journal Article https://pubmed.ncbi.nlm.nih.gov/35770859/	Every stimulant-containing xylazine-positive case also included an opioid. Stimulants were present in 53% of cases, cannabinoids in 30% and benzodiazepines in 26%. Xylazine's geographic distribution and prevalence grew during the study period.	N/A	450mg (injected) - for one case studied	Injection<br>Inhalation<br>Dermal Exposure<br>Ingestion	N/A	40
Journal Article https://injuryprevention.bmj.com/content/injuryprev/27/4/395.full.pdf	Between 2010 and 2015, xylazine was found in less than 2% of fatal heroin and/or fentanyl overdose cases; in 2019, it was found in 262 (31%) of the 858 cases of fatal heroin and/or fentanyl overdose. Of the 262 fatal cases, 76% were male. 100% of these fatal cases in 2019 were positive for fentanyl, as well as xylazine.	People stated euphoric effects lasted longer, like heroin before it was replaced with fentanyl	N/A	Injection	N/A	41
Journal Article https://www.sciencedirect.com/science/article/pii/S2772632023000582	In 2023, xylazine addiction has rapidly grown into a global concern and misuse has increased alarmingly. Serious repercussions have been seen in 2023 due to xylazine quickly growing into a global concern. Between 2019-2021, fatal overdoses in New York increased by more than 80%.	Drug of abuse, drug of sexual assault attempt, accidental/ intentional poisoning	N/A	Oral administration, inhaled, sniffed, injected	Online Source	50
Report https://www.cfsre.org/images/content/reports/public_alerts/Medetomidine_Public_Health_Alert__Final.pdf	A toxic adulterant alert sent out in December 2023 due to medetomidine/dexmedetomidine being identified as an adulterant in illicit drug material. Medetomidine (potent veterinary anaesthetic) has frequently been observed in samples containing fentanyl and	N/A	N/A	N/A	N/A	59

Type of Publication	Main Findings	Specifics - illicit Indication	Dose	Route	Source	Reference Number
	xylazine and also heroin and cocaine.					
Retrospective, Secondary Data Analysis https://pubmed.ncbi.nlm.nih.gov/36504413/	An increase of xylazine deaths in West Virginia have gone from 1% (2019) to 5% (2021). Deaths involving xylazine had more coin toxicants, compared to non-xylazine deaths. 98% of xylazine deaths involved fentanyl. There was a greater history of drug/alcohol use with xylazine decedents.	N/A	N/A	N/A	N/A	34
Journal Article https://pubmed.ncbi.nlm.nih.gov/37700329/	Xylazine-related overdoses in the United States have been escalating rapidly and show little indication of decelerating, posing a significant public health crisis. The 'speed-ball' mixture of heroin, cocaine and xylazine is obtainable for $8. Monthly rates of fentanyl mixed with xylazine overdose deaths increased nearly fourfold (from 2.9% to 10.9%) between January 2019 - June 2022.	N/A	N/A	Injection (84.5%), inhalation (14.1%), smoking (1.4%)	N/A	46
Report https://www.dea.gov/sites/default/files/2022-12/The%20Growing%20Threat%20of%20Xylazine%20and%20its%20Mixture%20with%20Illicit%20Drugs.pdf	The prevalence of xylazine is increasing although limited scientific research has been conducted on the effects of the drug in the body. The Centre for Disease Control and Prevention does not include xylazine-positive overdose deaths meaning it's prevalence is widely underestimated. A significant jump in xylazine deaths in the US from 2020-2021 has been reported. Northeast US has experienced a 103% increase, South - 1127% increase, Midwest - 516% increase and West - 750% increase.	N/A	N/A	N/A	N/A	58
Report https://www.cdc.gov/mmwr/volumes/72/wr/mm7226a4.htm	In 21 US jurisdictions, the monthly percentage of deaths involving xylazine in the context of illicitly manufactured fentanyl (IMF) increased by 276%, rising from 2.9% in January 2019 to 10.9% in June 2022.	N/A	N/A	N/A	N/A	35
Report https://blogs.cdc.gov/nchs/2023/06/30/7408/	Males were at least twice as likely to die from overdoses involving xylazine each year from 2018 to 2021. The highest rate of overdose deaths involving xylazine in 2021 were among the 35-44 age group.	N/A	N/A	N/A	N/A	56
Report https://www.dea.gov/alert/dea-reports-widespread-threat-fentanyl-mixed-xylazine	Public Safety Alert was announced in November 2022 warning the public of the increasing reports of fentanyl mixed with xylazine, stating that will be the deadliest drug threat the US has ever faced. The Drug Enforcement Administration reported the seizure of xylazine-fentanyl mixture in 48 of 50 states.	N/A	N/A	N/A	N/A	37

Type of Publication	Main Findings	Specifics - illicit Indication	Dose	Route	Source	Reference Number
Report https://www.emcdda.europa.eu/publications/european-drug-report/2023/drug-situation-in-europe-up-to-2023_en	Ketamine seizures remain high, often found in MDMA mixtures. The rise of 'Pink cocaine'—ketamine mixed with other synthetics—reflects growing consumer interest.	N/A	N/A	N/A	N/A	93
Report https://www.emcdda.europa.eu/publications/risk-assessments/ketamine_en	Hospitals, veterinary clinics and pharmaceutical distribution are ways ketamine is diverted for recreational use as sources have concluded the synthesis of ketamine as difficult. 12 deaths where ketamine had been identified occurred between 1987 and 2000. Concerns are present due to the 'near death' experiences and the unpredictability of the drug.	N/A	N/A	N/A	N/A	77
Report https://www.emcdda.europa.eu/publications/data-factsheet/syringe-residues-analysis-data-escape-project_en	Carfentanil was frequently identified in syringes from Vilnius (92%) and Riga (29%). Xylazine was found in 13% of syringes from Riga, often co-occurring with isotonitazene, metonitazene, or carfentanil.	N/A	N/A	N/A	N/A	136
Report https://www.emcdda.europa.eu/publications/european-drug-report/2023_en	Quantity of ketamine seized and reported to EU Early Warning System remains relatively high in recent years, suggesting it is consistently available in national drug markets, where it has been found in mixtures sold as 'pink cocaine'. A seized mixture in 2022 from Estonia included a mixture of protonitazene, metonitazene and xylazine.	N/A	N/A	N/A	N/A	43
Report https://www.emcdda.europa.eu/publications/european-drug-report/2023/injecting-drug-use_en	In Riga, Xylazine was found in 13% (25/194) of syringes. It was consistently mixed with isotonitazene or metonitazene in all 25 syringes and co-occurring with carfentil in 3 syringes. Carfentanil was commonly found in syringes from Vilnius (92%) and Riga (29%).	N/A	N/A	N/A	N/A	42
Report https://www.emcdda.europa.eu/publications/rapid-communication/new-psychoactive-substances-global-markets-glocal-threats-and-covid-19-pandemic_en	In 2019 there was 234 seizures of carfentanil (10044.2g). 17kg of new opioids were seized with 12kg being in the form of powders - 84% was carfentanil. In 2018, the total quantity to be seized was 1.9kg.	N/A	N/A	N/A	N/A	99
Report https://www.emcdda.europa.eu/publications/european-drug-report/2023/harm-reduction_en	Increasing polydrug consumption adds to the challenges of developing effective responses to reduce drug overdose deaths and drug-related poisonings. Mixtures containing novel benzodiazepines, novel opioids and the tranquiliser xylazine, has been reported in Estonia.	N/A	N/A	N/A	N/A	44
Report	Xylazine was identified in one fatality in	N/A	N/A	N/A	N/A	39

Type of Publication	Main Findings	Specifics - illicit Indication	Dose	Route	Source	Reference Number
https://www.emcdda.europa.eu/publications/european-drug-report/2023/drug-induced-deaths_en	2022.					
Report https://www.emcdda.europa.eu/news/2023/european-drug-report-2023-highlights_en	The increasing diversity in drug supply and usage poses novel challenges for drug policy and healthcare in Europe. The mixtures of novel benzodiazepines and opioids, with xylazine, has the potential to impact European health.	N/A	N/A	N/A	N/A	45
Report https://www.emcdda.europa.eu/ews25_en	In 2020, approximately 1.2 tonnes of seized material consisted mainly of aryl cyclohexylamines, with ketamine making up the vast majority at 1.1 tonnes (93%). In 2020, carfentanil made up 52% of opioid seizures. Argentina has reported the adulteration of cocaine with carfentanil, leading to deaths and non-fatal poisonings.	N/A	N/A	N/A	N/A	92
Report https://www.emcdda.europa.eu/publications/risk-assessments/carfentanil_en	Carfentanil is mainly seized as a powder but has been seen as a liquid, although in Europe it is typically administered via intravenous injection. Carfentanil misuse may be underreported due to not being part of most routine drug screening. There is limited information regarding the dose regimens of carfentanil and the abuse liability in humans.	N/A	N/A	N/A	N/A	100
Report https://www.emcdda.europa.eu/publications/european-drug-report/2023_en	Around 930 new psychoactive substances were being monitored by the EMCDDA by the end of 2022. Ketamine has gained prominence as a preferred drug among certain demographics.	N/A	N/A	N/A	N/A	78
Report https://www.emcdda.europa.eu/publications/edr/trends-developments/2022_en	Belgium and the Netherlands announced the dismantling of laboratories producing ketamine. 1600 seizures and 240kgs of ketamine was reported by 16 EU countries. 13% of people who used drugs in the last 12 months used ketamine, from the European Web Survey on Drugs.	N/A	N/A	N/A	N/A	79
Report https://www.emcdda.europa.eu/publications/edr/trends-developments/2021_en	Until 2021, there was inadequate monitoring of ketamine, which restricted the comprehension of its usage and its impact on public health. Denmark reported a last year prevalence of ketamine of 0.6% in 2017, and Romania 0.8% in 2019.	N/A	N/A	N/A	N/A	80
Report https://www.emcdda.europa.eu/publications/joint-reports/carfentanil_en	Until 2017, the EMCDDA had 755 seizures of carfentanil reported by seven Member States. Seizures reported carfentanil was mixed with other opioids or the synthetic	N/A	N/A	N/A	N/A	101

Type of Publication	Main Findings	Specifics - illicit Indication	Dose	Route	Source	Reference Number
	cathinone alpha-PHP. 48 deaths were reported to the EMCDDA up until 2017 - 85% were male and 15% female.					
Report https://www.wedinos.org/resources/downloads/Annual-Report-22-23-English.pdf	In 2022/2023, ketamine was the 5th most identified psychoactive. Ketamine was the 7th most intended purchased drug but was 6th most common drug identified post analysis. Ketamine was the second most common drug identified (206) from the 1112 samples analysed from 22 Nighttime Economy Venues and 2 festivals. 204 samples of ketamine were submitted during 2021-2022, with 6% of these containing no ketamine. The initial sample of xylazine was received in January 2020, followed by 10 subsequent samples containing xylazine. Among the 9 samples received between April 2022 and March 2023, none were submitted with xylazine listed as the intended purchase.	N/A	N/A	N/A	N/A	81
Report https://www.wedinos.org/resources/downloads/Annual-Report-21-22-English.pdf	Ketamine was the 4th most identified psychoactive substance. 213 samples of ketamine were identified from the 1102 samples from 24 Nighttime Economy Venues and 3 festivals - making it the second most identified substance after cocaine. 160 samples were submitted as ketamine, with 8% containing no ketamine.	N/A	N/A	N/A	N/A	82
Report https://www.wedinos.org/resources/downloads/Annual-Report-20-21-English.pdf	Ketamine was the 3rd most prevalent drug submitted by individuals aged 0-17 years. Ketamine was the 7th most intended purchased drug but was the 10th most common drug identified post analysis.	N/A	N/A	N/A	N/A	83
Report https://www.wedinos.org/resources/downloads/PHILTRE-AR-Eng-19-20.pdf	Ketamine was the 4th most identified psychoactive substance. Ketamine was the 6th most intended purchased drug but the 8th most common identified drug post analysis. From the 1048 samples received from Nighttime Economy and Festivals, ketamine was the 3rd most common drug identified, after cocaine and MDMA.	N/A	N/A	N/A	N/A	84
Report https://www.wedinos.org/resources/downloads/Annual_Report_201819.pdf	Ketamine was the 3rd most prevalent substance identified, after cocaine and MDMA. From the 339 samples identified from Nighttime Economy and Festivals, ketamine was the most prevalent drug.	N/A	N/A	N/A	N/A	85
Report https://www.wedinos.org/resources/downl	Ketamine was 5th most identified NPS. A sample submitted with intent to be ketamine	N/A	N/A	N/A	N/A	86

Type of Publication	Main Findings	Specifics - illicit Indication	Dose	Route	Source	Reference Number
oads/Philtre_Annual_Report_2017-18.pdf	was identified as beta-hydroxy fentanyl.					
Report https://www.wedinos.org/resources/downloads/Philtre_Annual_Report_2016-17.pdf	Ketamine was the 6th most identified NPS. Ketamine bought by users were analysed and sampled to be cocaine or furanylfentanyl.	N/A	N/A	N/A	N/A	87
Report https://www.wedinos.org/resources/downloads/WEDINOS_Annual_Report_2015-16_FINAL.pdf	Ketamine was a new entry and was the 3rd most identified NPS.	N/A	N/A	N/A	N/A	88
Journal Article https://pubmed.ncbi.nlm.nih.gov/20221861/	Levamisole, an anti-parasitic is used as an adulterant in a high percentage of cocaine samples. This may be because it is a bulky white powder, similar to cocaine. Other theories include to increase profit and the idea of levamisole adulterated cocaine effecting the ability to be detected by dogs/analytical methods. It was reported that levamisole was found to affect the endogenous opiate levels, including codeine and morphine.	Adulterant	N/A	N/A	N/A	131
Journal Article https://pubmed.ncbi.nlm.nih.gov/30485426/	In Kentucky, cocaine and methamphetamine were the main controlled substances and levamisole was the most prevalent adulterant detected (17.5%). Xylazine was present as a cutting agent in 4.6% of heroin samples, 11% of fentanyl samples and 2.6% of co-caine samples.	As an adulterant	N/A	N/A	N/A	132

PRISMA 2020 Checklist

Section and Topic	Item #	Checklist item	Location where item is reported
**TITLE**			
Title	1	Identify the report as a systematic review.	Title
**ABSTRACT**			
Abstract	2	See the PRISMA 2020 for Abstracts checklist.	See abstract checklist attached
**INTRODUCTION**			
Rationale	3	Describe the rationale for the review in the context of existing knowledge.	Introduction
Objectives	4	Provide an explicit statement of the objective(s) or question(s) the review addresses.	Introduction
**METHODS**			
Eligibility criteria	5	Specify the inclusion and exclusion criteria for the review and how studies were grouped for the syntheses.	Methods
Information sources	6	Specify all databases, registers, websites, organisations, reference lists and other sources searched or consulted to identify studies. Specify the date when each source was last searched or consulted.	Methods
Search strategy	7	Present the full search strategies for all databases, registers and websites, including any filters and limits used.	Methods
Selection process	8	Specify the methods used to decide whether a study met the inclusion criteria of the review, including how many reviewers screened each record and each report retrieved, whether they worked independently, and if applicable, details of automation tools used in the process.	Methods/Author contributions
Data collection process	9	Specify the methods used to collect data from reports, including how many reviewers collected data from each report, whether they worked independently, any processes for obtaining or confirming data from study investigators, and if applicable, details of automation tools used in the process.	Author contributions
Data items	10a	List and define all outcomes for which data were sought. Specify whether all results that were compatible with each outcome domain in each study were sought (e.g. for all measures, time points, analyses), and if not, the methods used to decide which results to collect.	Methods
	10b	List and define all other variables for which data were sought (e.g. participant and intervention characteristics, funding sources). Describe any assumptions made about any missing or unclear information.	No assumptions were made. Where data was unavailable, this was highlighted in supplementary information table by using 'N/A'.
Study risk of bias assessment	11	Specify the methods used to assess risk of bias in the included studies, including details of the tool(s) used, how many reviewers assessed each study and whether they worked independently, and if applicable, details of automation tools used in the process.	Methods
Effect measures	12	Specify for each outcome the effect measure(s) (e.g. risk ratio, mean difference) used in the synthesis or	N/A in this case

Section and Topic	Item #	Checklist item	Location where item is reported
		presentation of results.	
Synthesis methods	13a	Describe the processes used to decide which studies were eligible for each synthesis (e.g. tabulating the study intervention characteristics and comparing against the planned groups for each synthesis (item #5)).	Methods
	13b	Describe any methods required to prepare the data for presentation or synthesis, such as handling of missing summary statistics, or data conversions.	Methods
	13c	Describe any methods used to tabulate or visually display results of individual studies and syntheses.	Methods
	13d	Describe any methods used to synthesize results and provide a rationale for the choice(s). If meta-analysis was performed, describe the model(s), method(s) to identify the presence and extent of statistical heterogeneity, and software package(s) used.	Methods
	13e	Describe any methods used to explore possible causes of heterogeneity among study results (e.g. subgroup analysis, meta-regression).	N/A
	13f	Describe any sensitivity analyses conducted to assess robustness of the synthesized results.	N/A
Reporting bias assessment	14	Describe any methods used to assess risk of bias due to missing results in a synthesis (arising from reporting biases).	Methods
Certainty assessment	15	Describe any methods used to assess certainty (or confidence) in the body of evidence for an outcome.	N/A
**RESULTS**			
Study selection	16a	Describe the results of the search and selection process, from the number of records identified in the search to the number of studies included in the review, ideally using a flow diagram.	Results
	16b	Cite studies that might appear to meet the inclusion criteria, but which were excluded, and explain why they were excluded.	Results
Study characteristics	17	Cite each included study and present its characteristics.	Supplementary Information
Risk of bias in studies	18	Present assessments of risk of bias for each included study.	See attached table
Results of individual studies	19	For all outcomes, present, for each study: (a) summary statistics for each group (where appropriate) and (b) an effect estimate and its precision (e.g. confidence/credible interval), ideally using structured tables or plots.	N/A
Results of syntheses	20a	For each synthesis, briefly summarise the characteristics and risk of bias among contributing studies.	See attached table
	20b	Present results of all statistical syntheses conducted. If meta-analysis was done, present for each the summary estimate and its precision (e.g. confidence/credible interval) and measures of statistical heterogeneity. If comparing groups, describe the direction of the effect.	N/A
	20c	Present results of all investigations of possible causes of heterogeneity among study results.	N/A
	20d	Present results of all sensitivity analyses conducted to assess the robustness of the synthesized results.	N/A
Reporting biases	21	Present assessments of risk of bias due to missing results (arising from reporting biases) for each synthesis assessed.	Supplementary Information + See attached table
Certainty of evidence	22	Present assessments of certainty (or confidence) in the body of evidence for each outcome assessed.	N/A
**DISCUSSION**			
Discussion	23a	Provide a general interpretation of the results in the context of other evidence.	Discussion

Section and Topic	Item #	Checklist item	Location where item is reported
	23b	Discuss any limitations of the evidence included in the review.	Limitations
	23c	Discuss any limitations of the review processes used.	Limitations
	23d	Discuss implications of the results for practice, policy, and future research.	Conclusions
**OTHER INFORMATION**			
Registration and protocol	24a	Provide registration information for the review, including register name and registration number, or state that the review was not registered.	This work contributes to an Masters by Research Project at Swansea University
	24b	Indicate where the review protocol can be accessed, or state that a protocol was not prepared.	Protocol was not prepared
	24c	Describe and explain any amendments to information provided at registration or in thAse protocol.	N/A
Support	25	Describe sources of financial or non-financial support for the review, and the role of the funders or sponsors in the review.	Funding statement
Competing interests	26	Declare any competing interests of review authors.	Conflicts of Interest statement
Availability of data, code and other materials	27	Report which of the following are publicly available and where they can be found: template data collection forms; data extracted from included studies; data used for all analyses; analytic code; any other materials used in the review.	Supplementary Information Table

From: Page MJ, McKenzie JE, Bossuyt PM, Boutron I, Hoffmann TC, Mulrow CD, *et al*. The PRISMA 2020 statement: an updated guideline for reporting systematic reviews. BMJ 2021; 372: n71. doi: 10.1136/bmj.n71
